# The Vaginal Microbiota over an 8- to 10-Year Period in a Cohort of HIV-Infected and HIV-Uninfected Women

**DOI:** 10.1371/journal.pone.0116894

**Published:** 2015-02-12

**Authors:** Supriya D. Mehta, Brock Donovan, Kathleen M. Weber, Mardge Cohen, Jacques Ravel, Pawel Gajer, Douglas Gilbert, Derick Burgad, Greg T. Spear

**Affiliations:** 1 Division of Epidemiology and Biostatistics, University of Illinois at Chicago, School of Public Health, Chicago, Illinois, United States of America; 2 Department of Bioengineering, University of Illinois at Chicago, College of Medicine, Chicago, Illinois, United States of America; 3 The Core Center at Cook County Health and Hospital System, Chicago, Illinois, United States of America; 4 Institute for Genome Sciences, University of Maryland, and Department of Microbiology and Immunology, School of Medicine, University of Maryland, Baltimore, Maryland, United States of America; 5 Department of Immunology & Microbiology, Rush University Medical Center, Chicago, Illinois, United States of America; Fred Hutchinson Cancer Center, UNITED STATES

## Abstract

**Background:**

We identified predominant vaginal microbiota communities, changes over time, and how this varied by HIV status and other factors in a cohort of 64 women.

**Methods:**

Bacterial DNA was extracted from reposited cervicovaginal lavage samples collected annually over an 8–10 year period from Chicago Women’s Interagency HIV Study participants: 22 HIV-negative, 22 HIV-positive with stable infection, 20 HIV-positive with progressive infection. The vaginal microbiota was defined by pyrosequencing of the V1/V2 region of the 16S rRNA gene. Scheduled visits included Bacterial vaginsosis (BV) screening; clinically detected cases were referred for treatment. Hierarchical clustering identified bacterial community state types (CST). Multinomial mixed effects modeling determined trends over time in CST, by HIV status and other factors.

**Results:**

The median follow-up time was 8.1 years (range 5.5–15.3). Six CSTs were identified. The mean relative abundance (RA) of *Lactobacillus* spp. by CST (with median number of bacterial taxa) was: CST-1–25.7% (10), CST-2–27.1% (11), CST-3–34.6% (9), CST-4–46.8% (9), CST-5–57.9% (4), CST-6–69.4% (2). The two CSTs representing the highest RA of *Lactobacillus* and lowest diversity increased with each additional year of follow-up (CST-5, adjusted odds ratio (aOR) = 1.62 [95% CI: 1.34–1.94]; CST-6, aOR = 1.57 [95 CI: 1.31–1.89]), while the two CSTs representing lowest RA of *Lactobacillus* and higher diversity decreased with each additional year (CST-1, aOR = 0.89 [95% CI: 0.80–1.00]; CST-2, aOR = 0.86 [95% CI: 0.75–0.99]). There was no association between HIV status and CST at baseline or over time. CSTs representing lower RA of *Lactobacillus* were associated with current cigarette smoking.

**Conclusions:**

The vaginal microbial community significantly improved over time in this cohort of women with HIV and at high risk for HIV who had regular detection and treatment referral for BV.

## Introduction

Bacterial vaginosis (BV) is a clinical syndrome representing a shift in composition of the vaginal microbiota from *Lactobacillus* predominated to a more polymicrobial profile of strict and facultative Gram-negative anaerobes [1–2] The condition affects up to 30% of women in the general United States population [3]. BV in pregnant women increases risk of miscarriage, premature rupture of membranes, preterm birth, chorioamnionitis, post-abortal sepsis, and postpartum endometritis [4–7]. Women with BV also have increased risk of pelvic inflammatory disease [8–9] and acquisition of sexually transmitted infections [10–11]. A meta-analysis by Atashili et al. finds BV increases the risk of HIV acquisition by 60% (95% confidence interval 20–210%) [12], and prospective study by Cohen et al. found BV more than tripled the risk of HIV transmission from infected women to male sex partners (hazard ratio = 3.62; 95% confidence interval 1.74–7.52) [13]. Increased risk may stem from induction of local inflammation [14–16] and increased cervical HIV viral shedding [17–19]. In light of the frequency of BV and the associated adverse outcomes, modulation of the vaginal microbiota is increasingly recognized as a potential target in population level prevention of HIV and adverse pregnancy outcomes. Understanding how the vaginal microbiota promotes pathogenesis, which microbial species or community types represent potentially pathogenic states, and whether manipulation to reduce risk may be feasible or effective, requires a better understanding of the composition and ultimately function of the vaginal microbiota, and factors that may alter it.

Several studies published in the past several years have measured the vaginal microbiota via sequence analysis of 16S rRNA gene amplicons and examined factors associated with its composition or change in composition: menstrual cycle phase, menopausal stage, exogenous hormone exposure, douching, and HIV status. A previous cohort study by Jamieson et al. found greater odds of BV among HIV-positive women compared to HIV-negative women, and greater odds of BV infection among HIV-positive women with lower CD4 cell counts compared to women with HIV-positive women with higher CD4 cell counts [20]. While some longitudinal studies have found daily or weekly fluctuations in vaginal microbiota [21], Gajer et al. found community profiles that were stable, which could be either “good” (associated with persistently low Nugent score) or community profiles associated with consistently high Nugent score, hence at increased risk for adverse outcome [22]. Our study measured the vaginal microbiota community over several years of follow-up of HIV-infected and HIV-uninfected women enrolled in the Chicago Women’s Interagency HIV Study (WIHS) cohort. Our goal was to identify the predominant types of vaginal microbiota, how they varied by HIV status, and how vaginal microbiota changed over time.

## Methods

The study was approved by the Institutional Review Boards of Rush University Medical Center and the Cook County Health and Hospital systems. This study of the vaginal microbiota used data and biological specimens from the Chicago site of the WIHS, an ongoing prospective cohort study of United States women with and at risk for HIV infection. WIHS recruited women from 6 sites (Bronx and Brooklyn, New York; Chicago, Illinois; Los Angeles and San Francisco, California; and Washington, DC) during 3 phases (1994–1995; 2000–2001; 2012–2013) [23]. This study includes women enrolled in the initial (1994–1995) and second (2000–2001) WIHS recruitment waves. Details of recruitment and enrollment for WIHS have been described previously [23–25]. Written informed consent was obtained from all participants.

### Sample Selection

We identified three Chicago WIHS comparison groups based on HIV serostatus and disease progression and restricted our sample to those women who contributed at least 8 years of study observation with a minimum of 6 annual cervicovaginal lavage (CVL) repository samples available for testing from visits conducted between 1994 through 2010. We compared HIV negative women with HIV-positive women with stable infection and HIV-positive women with progressive infection. We examined differences in vaginal microbiota by rate of HIV progression because rapid HIV progression is associated with a more immunocompromised state, and these women may be prone to more diverse or pathogenic bacterial community profiles [20]. Regardless of antiretroviral use, HIV-positive subjects with stable infection were defined as those who had sustained CD4 cell counts >500 cells/mm^3^ over the observation period (allowing for one time point drop below 500 cells/mm^3^ as long as there was sustained rebound above 500 cells/mm^3^). HIV-positive women with progressive disease were defined as those with initial CD4 counts >500 cells/mm^3^ while not on therapy, followed by a drop to <200 cells/mm^3^, with a poor immunologic response to therapy (<100 cells/mm^3^ increase over 12 months) during the observation period. HIV uninfected (n = 25); HIV-infected with stable infection (n = 25); HIV infected with progressive disease (n = 25), were matched at the index visit for age, smoking status, sexual activity (frequency and number of partners), male condom and contraceptive use using MatchIt [26]. Exclusion criteria were: having gonorrhea, syphilis, trichomoniasis or chlamydia at the index visit; menopause (defined as sustained amenorrhea for at least 12 months with no subsequent menses) before age 40; having reported exchanging sex for drugs or money at more than 5 visits over the observation period.

### Specimen Collection and Processing

Cervicovaginal lavage samples, performed by irrigation of the cervix with 10 ml of nonbacteriostatic sterile saline, followed by aspiration from the posterior fornix were obtained during semi-annual WIHS study visits. CVL was processed generally within 4 hours of collection and 1 ml aliquots were frozen at -80° Celsius. CVL specimens from annual visits were retrieved from the Chicago WIHS repository, thawed, and centrifuged to pellet bacteria and bacterial DNA was isolated using the FastDNA Spin Kit for Soil (MP Biomedicals, Solon, OH) according to the manufacturer’s recommendation.

### PCR Amplification and Pyrosequencing of Barcoded 16S rRNA Gene V1–V2 Regions Amplicons

Pyrosequencing of barcoded 16S rRNA gene V1–V2 region amplicons was performed as described previously [27–28]. Universal primers 27F and 338R were used for PCR amplification of the V1–V2 hypervariable regions of 16S rRNA genes from genomic DNA extracts. The 338R primer included a unique sequence tag to barcode each sample. The primers were as follows: 27F-5’-GCCTTGCCAGCCCGCTCAGTC**AGAGTTTGATCCTGGCTCAG**-3’ and 338R-5’-GCCTCCCTCGCGC- CATCAGNNNNNNNNCAT**GCTGCCTCCCGTAGGAGT**-3’, where the underlined sequences are the 454 Life Sciences FLX sequencing primers B and A in 27F and 338R, respectively, and the bold letters denote the universal 16S rRNA primers 27F and 338R. The 8-bp barcode within primer 338R is denoted by 8 Ns. Barcoded amplicons were pooled in equimolar concentration and pyrosequenced on a Roche/454 Life Sciences FLX instrument. QIIME software [29] was used to bin the sequences based on their barcode, trim the primers and barcodes, remove sequences with homopolymeric runs longer than 8 bp, with ambiguous base calls and shorter than 100 bp. In addition, detection of chimeric sequences was performed using the UCHIME component of UCLUST [30] and chimeric sequences were removed. Taxonomic assignments were performed the Ribosomal Database Project (RDP) Classifier [31] trained on version 10 of the RDP database [32]. Each sample was run once since our past study indicates that duplicates of the PCR amplification and pyrosequencing steps have relatively low variability [27–28]. The program SpeciateIT was used to identify the species of *Lactobacillus* from the 16S rRNA gene sequences (website http://sourceforge.net/projects/speciateit/). From 581 specimens we obtained 3,749,105 high quality sequences. An average of 6,453 sequences were obtained from each sample (min = 169, max = 40,627).

### Measures

All sociodemographic and behavioral variables were measured by self-report during structured interviews administered by certified research assistants. Clinical variables were assessed by WIHS certified clinicians. Variables included age, current smoking status (yes vs. no), body mass index (BMI) category, parity, menopause, tubal ligation, hysterectomy, hormonal contraceptive use (any use, as well as oral hormonal contraception and Depo Provera), male condom use in the past 6 months (yes vs. no), and sexually active in the past 6 months and past 5 years. All explanatory variables were assessed as time varying covariates except for baseline HSV-2 seropositivity and stable or progressive HIV infection (which was classified based on the entire observation period). Highest educational attainment (assessed only at WIHS baseline), income, drug and alcohol use are provided as descriptive measures characterizing the population. Because 96% of women in the sample had been pregnant at some point during observation this was not examined as an explanatory factor. Date of last menstrual period (LMP) was available for 481 (94%) of 514 visits at which menopause or hysterectomy were not recorded. Among these, LMP more than 28 days prior to study visit was reported at 150 (31%) visits, with mean time of 51 days for those with LMP more than 28 days. Among these 150 visits, pregnancy was reported at 10. Due to the large proportion (~30%) of women whose menstrual phase could not be classified, we did not analyze menstrual stage as an independent variable. Hysterectomy was observed in 4 women (at 28 observations) and also was not examined as an explanatory variable. A clinical diagnosis of BV was evaluated at each visit, and was diagnosed if 3 of 4 Amsel’s criteria (pH>4.5 and clue cells positive and amine odor positive) were met. The pH of vaginal secretions was determined prior to cervico-vaginal lavage (CVL) collection, by adding genital fluid to indicator strips with a pH range of 4–7 (ColorpHast indicator strips, MCB Reagents, Gibbstown, NJ). Results of vaginal Gram stain were inconsistently available only through 2001, and therefore present for approximately one-third of observations (200 of 581 observations). For this reason, analysis of BV was limited to clinical diagnosis by Amsel’s criteria.

### Statistical Analyses

The goals of our analyses were to: (1) identify predominant vaginal bacterial community types, (2) examine whether the composition of vaginal bacterial communities differed by HIV status; (3) describe trends in the composition of vaginal microbiota over time; and (4) identify factors affecting the composition of the vaginal microbiota over time.

Hierarchical clustering was used to group observations with similar community types based on bacterial composition and relative abundances. An individual observation was assigned to a specific community state type (CST) at each time point measured. The Ward’s minimum variance linkage-criteria were used to perform hierarchical clustering on the complement of the Euclidean dissimilarity matrix. The number of clusters was determined by selecting the smallest number of groups so that there were no outliers (groups containing <5% of the sample). A heatmap was generated to visually demonstrate the relationship between average relative bacterial abundance and CST. Hierarchical clustering analyses and heatmap generation were performed using R environment [33]. The Shannon-Wiener index (log base *e*) was calculated to reflect bacterial diversity at the genus level for each observation using Primer 6.0 (Primer-E, version 6.1.13, United Kingdom).

To examine bacterial community composition by HIV status and over time we conducted mixed effects modeling with a binomial distribution and multinomial logit link, with subject-specific random slope and intercept. The multinomial outcome for analyses was community state type (CST); the CST with the lowest mean relative abundance of *Lactobacillus* was the base category, with each unit increase in category reflecting the next higher mean relative abundance of *Lactobacillus*. This analysis approach also enabled us to model other factors of interest influencing the vaginal microbiota composition and changes over time. Variables statistically significant at the p<0.10 level in univariate analyses were entered into multivariable analyses. Final model selection was assisted by examination of Akaike Information Criterion (AIC). Inferential analyses were conducted using SuperMix Version 1.1 (Scientific Software International, Lincolnwood, IL). To examine *Lactobacillus* species over time by HIV status we used a similar modeling approach as described above, but with a dichotomous outcome specifying the presence or absence of each particular species modeled. A sequence plot was generated to visually demonstrate CST over time for individuals (Stata/SE 13 for Windows, StataCorp, College Station, TX). A non-parametric test of trend was used to examine differences in subject characteristics by CST, and differences in *Lactobacillus* species by HIV status (Stata/SE 13).

## Results

Of the 75 subjects selected for inclusion in the study, CVL specimens and data meeting our inclusion and exclusion criteria were available for 22 HIV-negative women, 22 HIV-infected women with stable disease, and 20 HIV-infected women with progressive disease. Of the total 631 WIHS visits made by these 64 women, CVL specimens were available and 16S rRNA gene sequencing was completed for 581 (92%). The proportion of visits with missing 16S rRNA gene sequencing results was lowest for HIV-positive women with stable infection (3.7%) compared to HIV-negative (9.3%) and HIV-positive women with progressive disease (11.1%) (p = 0.012). All women in the analytic sample had at least 6 visits (range 6–11). The initial measurement in this study was also the first WIHS visit for 45 (70%) subjects, the second WIHS visit for 7 subjects, the third WIHS visit for 9 subjects, and the fourth WIHS visit for 3 subjects. The median follow-up time was 8.1 years [95% CI: 7.9–9.0]. The median time between visits was 0.97 years [95% CI: 0.96–0.98 years], reflecting our selection of annually collected specimens. The distribution of number of visits and follow-up time did not differ by subject HIV status. Subject characteristics at index visit by HIV status are shown in Table 1.

**Table 1:** Subject Characteristics at Initial Study Observation* by HIV-Status.

	HIV Negative,	HIV-Positive Stable,	HIV-Positive Progressive,	
Characteristic^1^	N = 22	N = 22	N = 20	P-value^
*Sociodemographics*				
Median age in years (95% CI)	28.5 (24.4–35.8)	33.9 (29.6–35.9)	31.0 (27.6–34.4)	0.163*
Educational attainment				0.507
Less than or some high school	6 (28.6)	12 (54.5)	9 (45.0)	
High school graduate	8 (38.1)	6 (27.3)	5 (25.0)	
Some college or above	7 (33.3)	4 (18.2)	6 (30.0)	
Annual Income				0.381
≤$18,000	16 (76.2)	19 (86.4)	19 (95.0)	
>$18,000–$36,000	4 (19.0)	3 (13.6)	1 (5.0)	
>$36,000–$75,000^+^	1 (4.8)	0 (0.0)	0 (0.0)	
*Behavioral Characteristics*				
Current smoker				0.167
No	7 (33.3)	4 (18.2)	9 (45.0)	
Yes	14 (66.7)	18 (81.8)	11 (55.0)	
Crack, cocaine, heroin use				0.158
Current	3 (14.3)	10 (45.5)	3 (15.0)	
Former	9 (42.9)	6 (27.3)	8 (40.0)	
Never	9 (42.9)	6 (27.3)	9 (45.0)	
Median number of drinks per week (95% CI)	0 (0–0.8)	1.5 (0–3)	0 (0–0.5)	0.081
Sexually active in the past 6 months				0.647
No	3 (14.3)	3 (13.6)	5 (26.0)	
Yes	18 (85.7)	19 (86.4)	15 (75.0)	
Male condom used in past 6 months				<0.001
No	10 (55.6)	1 (5.3)	1 (6.7)	
Yes	8 (44.4)	18 (94.7)	14 (93.3)	
*Clinical Characteristics*				
Median duration of HIV infection in years at baseline (95% CI)		1.0 (0–3.89)	2.0 (1–3.61)	0.382
Median CD4 cell count/mm^3^	1204	782	303	<0.001^ǂ^
(95% CI)	(982–1459)	(574–838)	(202–450)	
Median viral load (log 10)		3.31	4.17	0.020^ǂ^
(95% CI)		(2.77–3.71)	(3.39–4.58)	
Median Body mass index	33.8	26.9	23.2	<0.001^ǂ^
(95% CI)	(27.2–37.3)	(24.4–31.3)	(20.5–28.3)	
BMI category				0.075
18.5–24.9	3 (14.3)	8 (38.1)	9 (52.9)	
25.0–29.9	6 (28.6)	5 (23.8)	5 (29.4)	
≤ 30	12 (57.1)	8 (38.1)	3 (17.7)	
Median parity	2	2	1	0.629^ǂ^
(95% CI)	(1–4)	(1–3)	(1–1)	
Median days since last menstrual period	20.5	26	30	0.254^ǂ^
(95% CI)	(9.5–31)	(21–32.8)	(15.2–37.5)	
Tubal ligation				0.194
No	19 (90.5)	15 (68.2)	16 (80.0)	
Yes	2 (9.5)	7 (31.8)	4 (20.0)	
Any hormonal birth control use in past 6 months				0.044
No	14 (66.7)	21 (95.5)	17 (85.0)	
Yes	7 (33.3)	1 (4.5)	3 (15.0)	
Oral birth control pills in past 6 months^$^ ^#^				0.543
No	18 (85.7)	21 (95.5)	17 (85.0)	
Yes	3 (14.3)	1 (4.5)	3 (15.0)	
Depo-Provera in past 6 months^$^ ^#^				0.207
No	18 (85.7)	21 (95.5)	20 (100)	
Yes	3 (14.3)	1 (4.5)	0 (0.0)	
*Genitourinary conditions*				
HSV-2 infection				0.029
No	21 (100)	16 (72.7)	17 (85.0)	
Yes	0 (0.0)	6 (27.3)	3 (15.0)	
Candida infection				0.275
No	13 (61.9)	9 (40.9)	8 (40.0)	
Yes	8 (38.1)	13 (59.1)	12 (60.0)	
Median vaginal pH (95% CI)	5.0 (4.7–5.5)	5.0 (4.4–5.5)	5.5 (4.9–5.9)	0.320
Vaginal pH				0.912
4–4.5	3 (15.8)	4 (22.2)	3 (16.7)	
4.6–8	16 (84.2)	14 (77.8)	15 (83.3)	
Bacterial vaginosis detected by Amsel’s criteria	5 (22.7)	2 (9.1)	4 (20.0)	0.419
*Community State Type (CST)*				
CST 1	4 (18.2)	4 (18.2)	5 (25.0)	0.913
CST 2	9 (40.9)	8 (36.4)	9 (45.0)	
CST 3	4 (18.2)	4 (18.2)	4 (20.0)	
CST 4	2 (9.1)	5 (22.7)	2 (10.0)	
CST 5	1 (4.6)	0 (0.0)	0 (0.0)	
CST 6	2 (9.1)	1 (4.6)	0 (0.0)	

* Initial study observation refers to the current analysis rather than the initial WIHS visit.

^1^Cells may not sum to N due to missing data.

^^^Chi-square p-value is reported unless specified otherwise. Fisher’s exact test used when cell size <5.

^ǂ^ Kruskal-Wallis equality of populations test.

^+^ $75,000 is maximum income reported.

^$^ Oral contraception and Depo-Provera are both reported by the same woman.

^#^ Only 2 other types of hormonal birth control were reported. Ortho Evra and Norplant were reported at 3 and at 1 observation, respectively, and were not evaluated in analyses.

### Vaginal Microbial Community State Types

**Fig 1:**
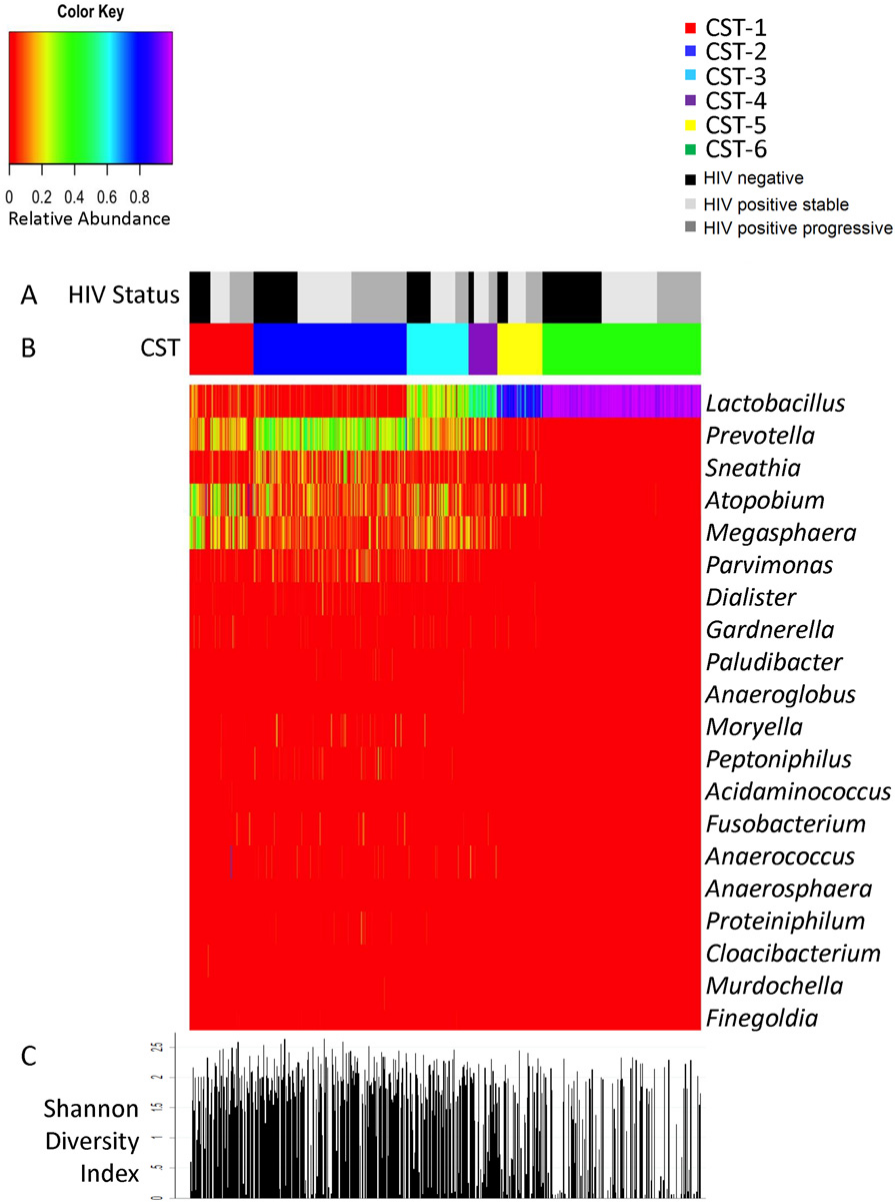
Heat map of bacterial relative abundance by Community State Type and HIV status. Heat map of relative bacterial abundance for 25 most commonly detected bacterial taxa by (A) community state type (CST) and (B) HIV status. (C) Shannon diversity index calculated for 581 observations.

Hierarchical clustering was used to group the 581 subject visits into community state types. The bacterial relative abundance of each of the 6 CSTs identified is shown in Fig. 1, and the presence and mean relative abundance are summarized in Table 2. The most common community types were CST-6 (n = 180, 31%) and CST-2 (n = 174, 30%), followed by CST-1 (n = 73, 13%), CST-3 (n = 70, 12%), CST-5 (n = 51, 8.8%), and CST-4 (n = 33, 5.7%). *Lactobacillus* was the most prevalent genus across all CSTs (detected in 78% of libraries), followed by *Prevotella* (64%), *Atopobium* (60%), *Megasphaera* (51%), and *Sneathia* (46%). CST-6 was of homogeneous composition (median of 2 bacterial taxa) with high mean RA (69.4%) of *Lactobacillus* spp. The mean RA of *Lactobacillus* spp. by CST (with median bacterial taxa) was as follows: CST-1–25.7% (10), CST-2–27.1% (11), CST-3–34.6% (9), CST-4–46.8% (9), CST-5–57.9% (4). While CST-1 and CST-2 both had similarly low mean RAs of *Lactobacillus*, *Lactobacillus* was more commonly detected among CST-1 (71.5%) samples than CST-2 (64.4%) samples. The mean RAs of the most abundant bacteria were similar for CST-1 and for CST-2, but the presence of *Sneathia*, *Parvimonas*, *and Dialister* was more common for CST-2 than CST-1. CST-3 (34.6%) and CST-4 (46.8%) had intermediate mean RAs of *Lactobacillus*, though the mean RA of CST-4 that was comprised of *Prevotella*, *Atopobium*, *Megasphaera*, and *Sneathia* was 28.8%, compared to 36.1% of the mean RA for observations classified as CST-3.

**Table 2:** Presence and Mean Relative Abundance of *Lactobacillus* species and 8 most abundant bacterial genera by Community State Type, HIV status, and Bacterial vaginosis status.

	Lactobacillus	L.iners*	L. crispatus*	L. gasseri*	L. jensenii*	Prevotella	Atopobium	Megasphaera	Sneathia	Parvimonas	Dialister	Gardnerella
CST-1 (n = 73)												
Present	71.2	94.2	38.5	23.1	15.4	78.1	76.7	67.1	49.3	52.0	46.6	32.9
Mean RA	25.7	81.3	5.9	8.3	3.9	16.3	9.4	9.7	3.6	2.9	1.1	0.9
CST-2 (n = 174)												
Present	64.4	92.0	43.8	22.3	20.5	80.5	74.7	66.1	66.7	66.1	56.9	33.3
Mean RA	27.1	80.7	11.6	4.6	2.1	18.4	7.9	6.2	6.0	2.8	1.3	0.8
CST-3 (n = 70)												
Present	80.0	92.9	33.9	19.6	17.9	74.3	74.3	67.1	57.1	57.1	40.0	32.9
Mean RA	34.6	79.5	11.8	2.2	6.1	15.4	9.8	6.8	4.2	3.0	0.9	0.8
CST-4 (n = 33)												
Present	84.8	100	28.6	17.9	28.6	75.8	69.7	60.6	48.5	54.5	36.4	30.3
Mean RA	46.8	87.5	7.5	3.9	0.1	11.5	7.5	7.0	2.8	1.7	1.0	1.0
CST-5 (n = 51)												
Present	92.2	93.6	27.7	27.7	19.1	58.8	54.9	41.2	31.4	29.4	35.3	11.8
Mean RA	57.9	81.7	5.8	10.2	1.5	10.3	5.3	4.2	2.1	1.2	0.9	0.2
CST-6 (n = 180)												
Present	87.2	72.6	52.9	52.9	38.9	36.7	33.3	24.4	23.9	20.0	17.8	16.7
Mean RA	69.4	55.3	28.6	8.5	6.4	6.9	5.0	2.8	2.0	0.8	0.3	0.4
HIV-negative (n = 186)												
Present	83.9	80.1	48.1	12.2	35.9	57.0	58.6	40.9	51.1	40.3	36.6	24.7
Mean RA	47.5	66.2	22.8	1.7	8.1	12.3	7.7	6.6	3.7	1.7	0.7	0.7
HIV-positive, stable n = 211)												
Present	79.6	88.7	42.3	26.2	27.4	64.9	64.5	50.7	55.9	46.4	38.4	25.6
Mean RA	45.4	77.9	15.6	3.9	2.1	13.4	7.2	5.3	3.7	2.0	1.0	0.6
HIV-positive, progressive (n = 184)												
Present	69.6	90.6	35.9	35.2	13.3	69.0	56.5	45.7	45.1	48.4	40.2	27.7
Mean RA	41.1	72.4	8.3	16.3	2.1	13.7	6.6	4.7	3.7	2.3	0.9	0.6
No BV by Amsel’s criteria (n = 405)												
Present	82.2	82.6	42.3	25.2	28.2	50.4	47.2	37.0	33.8	31.1	27.7	21.0
Mean RA	57.3	67.6	19.6	8.2	3.5	9.8	5.8	4.1	2.6	1.3	0.6	0.6
BV by Amsel’s criteria (n = 169)												
Present	66.3	99.1	43.8	20.5	17.9	98.2	98.2	86.4	76.9	80.4	65.9	38.5
Mean RA	12.7	88.2	5.3	1.6	4.4	21.7	10.7	9.2	6.5	3.7	1.6	0.8
All samples (n = 581)												
Present	77.8	86.5	42.5	23.9	26.3	63.7	60.1	51.0	46.0	45.1	38.4	26.0
Mean RA	44.7	56.3	12.4	5.2	3.2	13.1	7.2	5.5	3.7	2.0	0.9	0.6

All values are presented as percentages. RA = Relative Abundance.

*Reporting for *Lactobacillus* species is restricted to the 452 observations at which *Lactobacillus* was detected.

In keeping with the observed RA of *Lactobacillus* and other bacteria defining the CSTs, the proportion of women with clinically diagnosed BV was higher in CST-1 (42.5%), CST-2 (37.9%), and CST-3 (31.4%), and lower among women in CST-4 (27.3%), CST-5 (24.5%) and CST-6 (16.7%) (Table 3). Unsurprisingly, vaginal pH was increased in CSTs with lower RA of *Lactobacillus*: median vaginal pH was 5.8 in CSTs-1, -2, -3, decreasing to 5.3, 5.0, and 4.4 in CSTs-4, -5, and-6, respectively. Similarly, there was increasing detection of clue cells and whiff test positive results in CSTs with lower RA. Bacterial diversity (as reflected by median number of taxa detected and Shannon diversity index) was also increased in CSTs-4, -5, and-6 compared to CSTs-1, -2, and-3.

**Table 3:** Distribution of factors by bacterial community state type.

Characteristic^1^	CST 1, N^^^ = 73 n (%)	CST 2, N = 174 n (%)	CST 3, N = 70 n (%)	CST 4, N = 33 n (%)	CST 5, N = 51 n (%)	CST 6, N = 180 n (%)
HIV status						
HIV Negative	24 (32.9)	50 (28.7)	27 (38.6)	6 (18.2)	12 (23.5)	67 (37.2)
HIV positive, stable	22 (30.1)	61 (35.1)	28 (40.0)	17 (51.5)	20 (39.2)	63 (35.0)
HIV positive, progressive	27 (37.0)	63 (36.2)	15 (21.4)	10 (30.3)	19 (37.3)	50 (27.8)
Median age in years (95% CI) ^+^	35.3	35.1	34.8	36.0	38.6	39.5
	(32.3–36.6)	(33.5–35.9)	(33.9–37.1)	(32.7–38.5)	(35.9–42.6)	(37.8–40.7)
Current smoker^+^	45 (61.6)	140 (80.5)	58 (82.9)	26 (78.8)	35 (71.4)	96 (54.9)
BMI category^+^						
<24.9	29 (39.7)	71 (41.0)	20 (38.6)	9 (27.3)	16 (33.3)	48 (27.8)
25–29.9	17 (23.3)	56 (32.4)	24 (34.3)	13 (39.4)	15 (31.3)	55 (31.8)
> 30	27 (37.0)	46 (36.6)	26 (37.1)	11 (33.3)	17 (35.4)	70 (40.5)
Parity: 2 or more (vs. 0–1)^#^	51 (69.9)	117 (67.2)	53 (75.7)	25 (75.8)	36 (73.5)	137 (78.3)
Tubal ligation^+^	18 (24.7)	42 (24.7)	13 (19.1)	8 (24.2)	13 (28.9)	70 (40.0)
Oral birth control pills used in past 6 months	4 (5.5)	8 (4.7)	6 (8.6)	2 (6.1)	2 (4.2)	6 (3.4)
Depo-Provera used in past 6 months	3 (4.1)	16 (9.3)	4 (5.7)	1 (3.0)	3 (6.3)	9 (5.1)
Male condom used in past 6 months^+^	42 (57.5)	108 (62.8)	46 (65.7)	20 (60.6)	19 (39.6)	85 (48.6)
Number of sex partners in past 6 months^+^						
0	12 (16.7)	29 (17.1)	9 (13.2)	5 (15.6)	12 (26.1)	54 (31.4)
1	42 (58.3)	105 (61.8)	44 (64.7)	19 (59.4)	27 (58.7)	89 (51.7)
2 or more	18 (25.0)	36 (21.2)	15 (22.1)	8 (25.0)	7 (15.2)	29 (16.9)
Median vaginal pH (95% CI) ^+^	5.8 (5.5–5.8)	5.8 (5.5–5.8)	5.8 (5.5–5.8)	5.3 (4.8–5.8)	5.0 (4.5–5.5)	4.4 (4.4–4.6)
Clue cells detected^+^	35 (48.0)	75 (43.1)	23 (32.9)	11 (33.3)	15 (30.6)	37 (21.1)
Whiff test positive^+^	35 (48.0)	77 (44.3)	30 (42.9)	11 (33.3)	16 (32.7)	36 (20.6)
Two or more Amsel criteria^+^ $	39 (53.4)	85 (48.9)	31 (44.3)	13 (40.6)	19 (38.8)	41 (23.6)
Bacterial vaginosis^+^	31 (42.5)	66 (37.9)	22 (31.4)	9 (27.3)	12 (24.5)	29 (16.7)
Median number of bacterial genera^+^ (95% CI)	10 (9–12)	11 (11–12)	9 (7.3–12)	9 (6.0–10.7)	4.0 (3.0–8.0)	2.0 (1.0–2.0)
Median Shannon diversity index^+^ (95% CI)	1.8 (1.6–2.0)	1.9 (1.8–2.0)	1.7 (1.6–1.9)	1.4 (0.8–1.9)	0.7 (0.4–1.5)	0.1 (0.0–0.2)

^1^ Figures may not sum to N due to missing values.

^^^ N represents observations. CI = Confidence Interval

p-value by non-parametric test of trend:

^+^ = p-value <0.01;

^#^ = 0.01 < p-value <0.05;

^$^ At least two of the three criteria: Clue cells detected, whiff test positive, vaginal pH greater than 4.5.

### Community State Types by HIV Status

The distribution of CST did not differ by HIV status at baseline (Table 1). In modeling, compared to HIV negative women, there was no statistically significant association between HIV status and CST in time-adjusted (Table 4) or multivariable (Table 5) analyses. Among HIV positive women, duration of HIV infection at baseline was not associated with any community type (results not shown). In keeping with this, HIV status also was not associated with BV at initial study observation (Table 1) or over time (results not shown).

**Table 4:** Results of mixed effects multinomial modeling*: Time-adjusted crude odds of community state type by independent factors.

	CST 2	CST 3	CST 4	CST 5	CST 6
	OR [95% CI]	OR [95% CI]	OR [95% CI]	OR [95% CI]	OR [95% CI]
HIV status (vs. HIV Negative)					
HIV positive, stable	1.43 [0.69–2.95]	1.12 [0.50–2.49]	2.97 [0.97–9.12]	1.43 [0.40–5.12]	0.75 [0.16–3.47]
HIV positive, progressive	1.24 [0.81–1.02]	0.46 [0.19–1.11]	1.32 [0.40–4.39]	0.90 [0.24–3.35]	0.34 [0.08–1.51]
Age in years	1.01 [0.97–1.07]	1.04 [0.99–1.10]	1.04 [0.97–1.11]	1.08 [1.01–1.15]^+^	1.11 [1.04–1.18]^$^
Current smoker (Yes vs. No)	2.70 [1.46–5.01] ^$^	3.35 [1.49–7.54]^$^	2.52 [0.94–6.75]	1.25 [0.46–3.39]	0.69 [0.25–1.91]
BMI category (vs. <25)					
25–29.9	1.35 [0.67–2.74]	2.32 [0.98–5.50]	2.78 [0.96–8.05]	2.11 [0.71–6.30]	3.75 [1.26–11.2] ^+^
> 30	0.70 [0.36–1.34]	1.40 [0.63–3.10]	1.32 [0.47–3.72]	1.42 [0.50–4.04]	1.99 [0.67–5.95]
Parity (2 or more vs. 0–1)	1.00 [0.51–1.97]	1.34 [0.62–2.87]	1.30 [0.49–3.42]	0.69 [0.19–2.52]	0.94 [0.16–5.35]
Tubal ligation (Yes vs. No)	1.18 [0.59–2.38]	0.73 [0.32–1.65]	0.95 [0.36–2.53]	0.71 [0.24–2.10]	1.34 [0.41–4.37]
Oral birth control pills (Yes vs. No)	0.82 [0.23–2.93]	1.36 [0.36–5.16]	0.93 [0.16–5.52]	1.08 [0.15–7.94]	0.42 [0.06–3.10]
Depo-Provera (Yes vs. No)	2.10 [0.58–7.63]	1.33 [0.28–6.26]	0.73 [0.07–7.46]	4.91 [0.77–31.2]	5.43 [0.97–30.5]
Male condom use in past 6 months (Yes vs. No)	1.13 [0.63–2.02]	1.39 [0.70–2.78]	1.16 [0.49–2.72]	0.76 [0.32–1.79]	1.24 [0.54–2.84]
Number of sex partners in past 6 months (vs. 0)					
1	0.99 [0.45–2.15]	1.53 [0.58–4.09]	1.19 [0.36–3.91]	0.56 [0.18–1.71]	0.52 [0.18–1.50]
2 or more	0.83 [0.34–2.02]	1.14 [0.37–3.50]	1.09 [0.28–4.19]	0.32 [0.08–1.30]	0.34 [0.10–1.18]
Time in years	0.90 [0.80–1.01]	0.90 [0.78–1.04]	0.94 [0.79–1.12]	1.74 [1.46–2.07]^$^	1.78 [1.53–2.08] ^$^

*Reference category is Community State Type 1.

Each model is adjusted only for time.

OR = Odds Ratio; CI = Confidence Interval.

^$^ p<0.01;

^+^p<0.05.

**Table 5:** Results of mixed effects multinomial modeling*: Multivariable^ adjusted odds of Community State Type.

	CST 2	CST 3	CST 4	CST 5	CST 6
	OR [95% CI]	OR [95% CI]	OR [95% CI]	OR [95% CI]	OR [95% CI]
HIV status (vs. HIV Negative)					
HIV positive, stable	1.13 [0.54–2.36]	0.81 [0.35–1.92]	2.51 [0.78–8.12]	1.59 [0.42–5.96]	0.72 [0.18–2.90]
HIV positive, progressive	1.14 [0.56–2.32]	0.46 [0.19–1.13]	1.41 [0.42–4.75]	1.32 [0.32–5.50]	0.40 [0.08–1.96]
Age in years	1.02 [0.97–1.07]	1.04 [0.99–1.10]	1.02 [0.95–1.10]	1.08 [0.99–1.18]	1.15 [1.04–1.26] ^$^
Current smoker (Yes vs. No)	2.62 [1.39–4.95]^$^	3.12 [1.37–7.08] ^$^	2.23 [0.81–6.10]	1.34 [0.47–3.83]	0.69 [0.25–1.95]
Time in years	0.89 [0.80–1.00]	0.86 [0.75–0.99]^+^	0.92 [0.77–1.10]	1.62 [1.34–1.94] ^$^	1.57 [1.31–1.89] ^$^

*Reference category is community type 1.

^The model is adjusted for all variables presented.

OR = Odds Ratio; CI = Confidence Interval.

^$^ p<0.01;

^+^p<0.05.

### Trends Over Time in Community State Types

**Fig 2:**
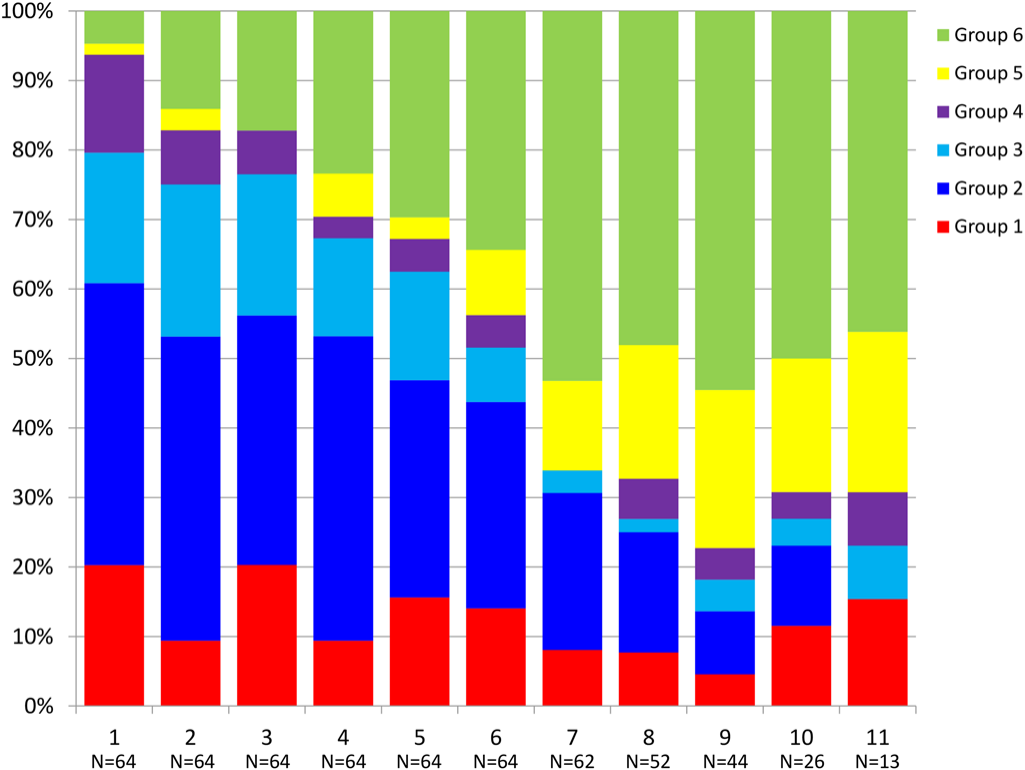
Proportion of visits with bacterial community state type over annual study observation.

**Fig 3:**
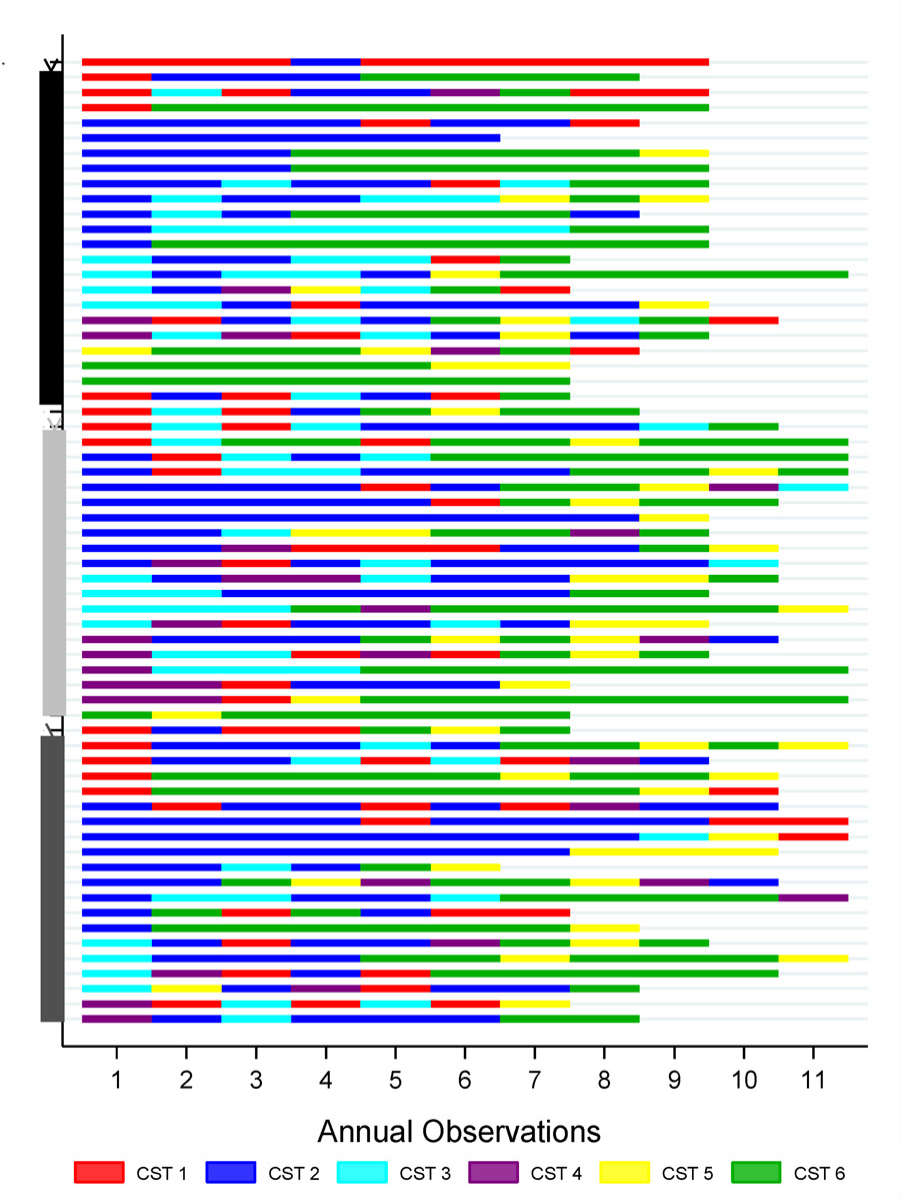
Sequence plot of community state type over annual study visit by individual. Follow-Up VisitCommunity state type (CST) for each individual over time. CST is indicated by color scheme in legend. HIV status is indicated by vertical bars on y-axis: black represents HIV-negative individuals; light grey represents HIV-positive individuals with stable infection; dark grey represents HIV-positive individuals with progressive infection.

Over follow-up time there was an increase in CST-6, the CST with the highest abundance of *Lactobacillus*, from 5% of observations at baseline to 30–50% of observations occurring at later follow-up visits (Fig. 2). CST-5 increased from 3% of observations at baseline to approximately 20% of observations at 8^th^ through 11^th^ annual microbiota assessments. CST-2 and CST-3, CSTs with relatively lower abundance of *Lactobacillus* and greater diversity, decreased over time. In multivariable adjusted analyses (Table 5), with each increasing year of follow-up time, compared to having a CST-1, the odds of CST-2 decreased by 11% [adjusted odds ratio (aOR) = 0.89, 95% CI: 0.80–1.00] and the odds of CST-3 decreased by 14% [aOR = 0.86; 95% CI: 0.75–0.99]. Conversely, with each additional year of follow-up time, the odds of CST-5 increased by 62% [aOR = 1.62; 95% CI: 1.34–1.94] and by 57% for CST-6 [aOR = 1.57; 95% CI: 1.31–1.89]. The sequence plot (Fig. 3) reflects at the individual level the general shift over time towards CSTs with greater relative abundance of *Lactobacillus* spp.

### Factors Associated with Community State Type

In time-adjusted analyses (Table 4), there were significant associations between increasing age and greater odds of CST-5 and CST-6 membership, while current smoking was associated with an increased odds of CST-2 and CST-3 (lower RA *Lactobacillus* CSTs). Parity, tubal ligation, and male condom use were not associated with any community type. In multivariable time-adjusted analyses (Table 5), increasing age remained statistically significantly associated with increasing odds of CST-5 and CST-6, while smoking remained a risk factor for CST-2 and CST-3. There were no meaningful or statistically significant interactions by time with HIV status or any other covariates.

### Lactobacillus Speciation by HIV Status, Community State Type, and BV Status

*Lactobacillus* was detected with relative abundance >1% in 452 (77.8%) specimens. Among these 452 specimens, 4 species accounted for >98.8% of the median relative abundance of *Lactobacillus* detected: *L*. *iners*, *L*. *crispatus*, *L*. *gasseri*, *L*. *jensenii* (Table 2). In addition to these 4, 10 other *Lactobacillus* species were detected: *L*. *coleohominis*, *L*. *vaginalis*, *L*. *rhamnosus*, *L*. *mucosae*, *L*. *spp*,, and 5 unclassified operational taxonomic units of *Lactobacillus*. Overall, the median number of *Lactobacillus* species detected per observation was 4.2 (range 1–12) and did not change over time (non-parametric test of trend, p = 0.36).

The presence of *L*. *iners* was lower (80.1%) among HIV negative observations than HIV positive observations (88–90%, Table 2). The presence of *L*. *crispatus* decreased from 48.1% among HIV negative observations to 42.3% among observations with HIV positive stable infection and 35.9% among observations with HIV positive progressive infection. The presence of *L*. *gasseri* was increased for HIV positive stable (26.2%) and HIV positive progressive (35.2%) observations compared to HIV negative observations (12.2%), while the presence of *L*. *jensenii* was lowest for HIV positive progressive observations (13.3%) compared to HIV negative observations (33.9%). Overall group differences in the RA of *L*. *iners* and *L*. *crispatus* by HIV status were not statistically significant in time-adjusted repeated measures analyses, though the increases in *L*. *gasseri* for HIV positive observations compared to HIV negative observations were statistically significant, as was the decreased presence of *L*. *jensenii* for HIV positive progressive observations compared to HIV negative observations (Table 6). The distribution of *Lactobacillus* species was most noticeably different for CST-6 (Table 2), which has the lowest prevalence of *L*. *iners* (RA 72.6% compared to 92–100% for CSTs 1–5), the highest prevalence of *L*. *crispatus* (52.9% compared to 28–44% for CSTs 1–5), the highest prevalence of *L*. *gasseri* (52.9% compared to 18–28% for CSTs 1–5), and highest prevalence for *L*. *jensenii* (39% compared to 15–29% for CSTs 1–5). Compared to women without BV (Table 2), for women with clinically detected BV there was a greater presence of *L*. *iners* (99% versus 83%) and lower prevalence of *L*. *jensenii* (18% versus 28%).

**Table 6:** Results of mixed effects binomial modeling: Time-adjusted crude odds of *Lactobacillus* species cluster among 452 samples with *Lactobacillus* detected.

	L. iners	L. crispatus	L. gasseri	L. jensenii
	aOR [95% CI]	OR [95% CI]	OR [95% CI]	OR [95% CI]
HIV status (vs. HIV Negative)				
HIV positive, stable	3.41 [0.55–21.1]	0.75 [0.40–1.43]	3.38 [1.09–10.4] ^$^	0.55 [0.17–1.77]
HIV positive, progressive	4.81 [0.68–34.0]	0.58 [0.29–1.16]	5.82 [1.85–18.9]^+^	0.19 [0.05–0.69]^$^
Time in years	1.09 [0.97–1.23]	1.00 [0.94–1.06]	0.92 [0.84–1.00]*	1.06 [0.97–1.16]

Model are adjusted for time. aOR = Odds Ratio; CI = Confidence Interval.

* The corresponding p-value is 0.0596.

^+^ p<0.01;

^$^ p<0.05.

## Discussion

Our study provides a unique and new perspective; we were able to examine the trajectory of the vaginal microbiota assessed annually over a median of 8.1 years in 64 women. Over time, the likelihood of having a *Lactobacillus* dominated vaginal microbiota increased regardless of HIV status, with statistically significant increases over time in community state types with low bacterial diversity and high RA of *Lactobacillus*, and concomitant decreases in CSTs with high diversity and low RA of *Lactobacillus*. It is unknown which is of more importance to risk of HIV, STI, or adverse pregnancy outcomes—transient shifts in the vaginal microbiota or persistently altered community state types—or how this varies by disease. For example, risk of adverse pregnancy outcomes versus increased risk of HIV acquisition or transmission may stem from different CSTs and different durations of altered microbiota. Understanding the role of the vaginal microbiota in relation to multiple disease outcomes will help define whether particular community state types are associated with increased risk outside of a definition based primarily on BV.

We did not find any differences in vaginal microbial community type by HIV status. BV is a risk factor for HIV transmission and acquisition [14–19], but longitudinal studies examining how HIV *impacts* the vaginal microbiota are not as readily available. In our previous study, Spear et al. found that HIV-infected women with BV had greater microbial diversity than HIV-negative women with BV, but that there were no community differences between HIV positive and HIV negative women who were BV negative by Nugent scoring of Gram stain [34]. A previous analysis of the entire WIHS sample by Watts et al. also did not find an association between HIV status and BV diagnosed by Nugent scoring of Gram stain at baseline or over follow-up, and observed significant declines in BV for HIV-negative and HIV-positive women [35]. These findings are in contrast to those who observe higher rates of BV among HIV-positive women compared to HIV-negative women [20, 36]. These different results may stem from population differences in measure of BV, immune status, medication use, race, health care access, sexual behavior, study design or many other factors. Despite these differences, the results of Schellenberg et al. also demonstrate a decline over time in the rate of BV, and they also posit that the improved vaginal health could stem from repeated assessment and treatment, or reduced behavioral risks over time [36].

We found (1) that the RA of *L*. *iners* was lower among observations classified as CST-6; (2) that clinically diagnosed BV was less common among vaginal microbiomes of CST-6; and (3) increasing RA of *L*. *iners* was associated with increasing odds of clinically detected BV. We found that compared to HIV negative women, HIV positive women were more likely to have *L*. *gasseri*, which increased among observations with BV. The occurrence of *L*. *jensenii* was lower for observations at which BV was detected, and we also observed a decrease in occurrence of *L*. *jensenii* for observations with progressive HIV infection. Despite finding associations between HIV status and *Lactobacillus* species that were also associated with BV, we did not find an association between HIV status and BV, which may be due to limited power to detect modest associations. Alternatively, unmeasured aspects of the vaginal microbiome, such as community function or host immune response, may compensate for microbiome alterations that do not produce clinical disease differences. In a study comparing the vaginal microbiome between HIV positive and HIV negative women, Schellenberg et al. found a non-statistically significant lower occurrence of *L*. *crispatus* among 12 HIV positive women (8%) compared to 32 HIV negative women (59%) [37]. *L*. *crispatus* was detected in a much higher percentage (36–42%, Table 2) of our HIV-positive samples. These results highlight the challenges of comparing vaginal microbiomes between different populations. Viewed positively, these discrepancies raise new questions, such as to what extent the subject characteristics (race, age, environment, sexual behavior, STIs, genital hygiene, immune status, health care access, etc.) modify the vaginal microbiome.

We identified 6 community types representing the vaginal microbiota in this population. Given the variability between subjects that clustered within our CSTs, we agree with the conclusion by Ravel *et al*. that core microbiota are not defined solely by the bacterial taxa within the community, but likely—at least in part—through microbial community functions [28]. While our study was not designed to evaluate this, we did find phenotypic differences by CST. Women with observed CSTs of higher species diversity and lower RA of *Lactobacillus* were more likely to also be diagnosed with BV clinically and vaginal pH was also elevated (Table 3). We did not include *Lactobacillus* species in the hierarchical clustering analysis that was used to determine CSTs. These species are important determinants of BV status, with *L*. *gasseri* [28] and *L*. *iners* [30] being more abundant in vaginal microbiota with elevated Nugent scores, and *L*. *crispatus* being more abundant in vaginal microbiota with low Nugent scores [38]. Including these species in the determination of the clusters would influence the cluster generation toward discrimination between BV-type and BV-free status, rather than to examine how microbiota more broadly varies by HIV status. Therefore, we did not include the *Lactobacillus* species in the determination of CSTs.

Among our cohort of Black women, vaginal pH was elevated (>4.5) at 68% of observations. In a study of 396 asymptomatic, sexually active women [28], vaginal pH varied significantly by race, with Black women having a median vaginal pH of 4.7, significantly higher than the median of 4.2 for White women. Historically, normal vaginal pH is considered to be in the range of 3.5–4.5. In the development of the initial criteria for non-specific vaginitis by Amsel et al., the threshold of 4.5 for “elevated” vaginal pH was based on comparison of vaginal pH against other symptoms [39]. Elevated pH was detected in 33% of “normal” women and normal pH (<4.5) in 19% of women with vaginitis. The race of participants is not reported in this article, but if based on a primarily White patient population, the results and normative vaginal pH may not be generalizable to women of other race.

As reflected in Fig. 1 and Table 2, *Gardnerella* was the 8^th^ most predominant genus, and present in 26% of libraries: 38% at observations meeting Amsel’s criteria for BV and 21% at observations without clinically diagnosed BV. While some studies find high prevalence of *Gardnerella* in women without BV [40–42], our prevalence is comparable to findings by Schwebke et al., who found that *G*. *vaginalis* was detected by PCR in only 38.5% of women with normal Nugent scores [43]. Previous characterization of the vaginal microbiome of 382 asymptomatic reproductive age women by Ravel et al. found similarly low relative abundance of *Gardnerella* [28].

Other prospective studies [7, 44] observe increased risk of BV among women who smoke cigarettes. In a pilot study by Brotman et al., women who smoked cigarettes were statistically significantly more likely to have a low-*Lactobacillus* vaginal community and higher Nugent scores [45]. Women who smoke cigarettes demonstrate down-regulated systemic inflammatory immune response, including that of the cervix, which may lead to an environment that is more permissive of pathogenic bacteria [46]. Additionally, chemicals from cigarettes have been recovered from vaginal secretions, and demonstrated *ex vivo* to increase phage induction that may reduce levels of *Lactobacillus* [47]. Alternatively, although there are biologically plausible mechanisms, the observed association may be a result of confounding through correlation of smoking with multiple sexual risk behaviors [45, 48] that this analysis was unable to adjust for. The previous WIHS analysis by Watts *et al*. also observed increased risk of BV associated with cigarette smoking, and a protective effect of increasing age [35].

A systematic review by Van de Wijgert *et al*. reports that oral contraception and DMPA use reduce risk of BV by 10–20% and 18–30%, respectively [49]. Of the 4 studies in the review that included measure of the vaginal microbiota composition, increasing estrogen levels were associated with community types dominated by *Lactobacillus* species. The lack of association we observed between hormonal contraceptive use and vaginal microbiota types was unexpected, and may have been due to limited power or low adherence among OC users. A meta-analysis by Fethers et al. of 28 world-wide studies estimated a 20% protective effect of condom use on BV [50]. We did not find a protective association between male condom use and CSTs with higher rates of BV, though this may have been due to lack of specificity in the measure of condom use analyzed.

The women in the cohort were enrolled in the WIHS and attended semi-annual visits that included vaginal examinations with assessment of BV by Amsel’s criteria, and referral for treatment if indicated. Women with symptomatic and asymptomatic BV may have been treated. For general population women who seek medical attention for symptomatic BV, treatment often does not lead to sustained improvement and BV recurs within 3–6 months in up to 30% of women [51]. Our results also suggest that regular detection and treatment of BV regardless of symptoms could be of benefit. Little data are available on the clinical course of asymptomatic women with altered vaginal bacterial communities. In an observational study of women with asymptomatic BV following treatment of symptomatic BV, 37% developed symptoms by 3–4 months [52]. In women at high risk for BV, regular screening for BV (regardless of symptoms) could be an important component of primary care if it leads to sustained improvements in the vaginal microbiota—i.e., CSTs that are associated with reduced risk of BV, adverse pregnancy outcomes, and HIV or STI acquisition or transmission.

### Limitations

The WIHS cohort provided a unique opportunity to examine the composition of the vaginal microbiota over a 7–12 year period with comprehensive measures of behavioral and clinical data; the strength of the study is somewhat countered by limited generalizability. Because of our exclusion and matching criteria, our sample is not representative of the Chicago WIHS cohort and is likely healthier overall, given our requirement for at least 8 years of study observation. With use of primer sets 27F and 338R, *G*. *vaginalis* could be underrepresented in this study, and as a consequence *Atopobium vaginae* could be overrepresented; however, there are known biases with any primer set [53]. Community state type is a summative description of the vaginal microbial environment. However, the goal of the current analysis was not to examine whether individual species were causative to a *disease* outcome, but whether the bacterial *community*, and changes in the bacterial community over time, differed by HIV status. The associations and magnitude of measures identified in this sample may be biased by selection, and should be interpreted with caution. Imprecise matching resulted in residual confounding, as indicated by statistically significant baseline differences in male condom use and contraceptive use (Table 1). We addressed this residual confounding through adjusted analyses, though the matching process itself obscures the associations and magnitudes of association. It is possible that decreases over time in unmeasured sexual risk behaviors could have led to the increase in vaginal communities with lower diversity and higher abundance *Lactobacillus*. The lack of association of CST with expected variables—such as hormonal oral and injectable contraceptive methods and condom use—may have resulted from regular screening for BV with high likelihood of treatment. Our definition of BV was based on modified Amsel’s criteria, which did not consider vaginal discharge. Therefore, we likely underestimated the prevalence of BV, which may have attenuated power or measures of association. We did not have a measure of treatment for BV in our database, and our inference regarding the effect of screening on CST over time must be taken with caution. Our definition of stable and progressive HIV infection was based on CD4 cell count rather than viral load and/or clinical outcomes [54], but is similar to that used by others [55].

## Conclusions

HIV status was not associated with vaginal microbial community composition at baseline or over 8–10 years of follow-up in this regularly followed, special cohort of HIV-positive and high risk HIV-negative women.
